# K-Promoted Ni-Based Catalysts for Gas-Phase CO_2_ Conversion: Catalysts Design and Process Modelling Validation

**DOI:** 10.3389/fchem.2021.785571

**Published:** 2021-11-18

**Authors:** J. Gandara-Loe, E. Portillo, J. A. Odriozola, T. R. Reina, L. Pastor-Pérez

**Affiliations:** ^1^ Department of Inorganic Chemistry and Materials Sciences Institute, University of Seville-CSIC, Seville, Spain; ^2^ Chemical and Environmental Engineering Department, School of Engineering, University of Seville, Sevilla, Spain; ^3^ Department of Chemical and Process Engineering, University of Surrey, Guildford, United Kingdom

**Keywords:** CO_2_ valorisation, alkali promoters, Ni-based catalysts, RWGS, low temperature, potassium

## Abstract

The exponential growth of greenhouse gas emissions and their associated climate change problems have motivated the development of strategies to reduce CO_2_ levels via CO_2_ capture and conversion. Reverse water gas shift (RWGS) reaction has been targeted as a promising pathway to convert CO_2_ into syngas which is the primary reactive in several reactions to obtain high-value chemicals. Among the different catalysts reported for RWGS, the nickel-based catalyst has been proposed as an alternative to the expensive noble metal catalyst. However, Ni-based catalysts tend to be less active in RWGS reaction conditions due to preference to CO_2_ methanation reaction and to the sintering and coke formation. Due to this, the aim of this work is to study the effect of the potassium (K) in Ni/CeO_2_ catalyst seeking the optimal catalyst for low-temperature RWGS reaction. We synthesised Ni-based catalyst with different amounts of K:Ni ratio (0.5:10, 1:10, and 2:10) and fully characterised using different physicochemical techniques where was observed the modification on the surface characteristics as a function of the amount of K. Furthermore, it was observed an improvement in the CO selectivity at a lower temperature as a result of the K-Ni-support interactions but also a decrease on the CO_2_ conversion. The 1K catalyst presented the best compromise between CO_2_ conversion, suppression of CO_2_ methanation and enhancing CO selectivity. Finally, the experimental results were contrasted with the trends obtained from the thermodynamics process modelling observing that the result follows in good agreement with the modelling trends giving evidence of the promising behaviour of the designed catalysts in CO_2_ high-scale units.

## Introduction

The rapid development of the global economies joined by the population growth has fuelled the emission of greenhouse gases, placing our planet in a limit situation. Inherently, the emission of these gases has caused the increase of the Earth temperature, which is responsible for multiple natural phenomena. (Jetten et al., 2021) Furthermore, in 2020, the coronavirus pandemic has forced to stop human activities, partially lowering the CO_2_ levels. However, the necessity of high amounts of chemicals and the large-scale fabrication of devices to fight the pandemic have boosted the emission of CO_2_. COVID-19 pandemic is a reminder that there is a link between climate change issues and certain types of diseases spread. (Letcher, 2021) For instance, it has been reported a direct relationship between the population density, human encroachment on natural areas, and the dissemination of zoonotic diseases. (National Research Council (US), 2009) All of these have motivated the development of strategies not only in the replacement of conventional energy sources but also to mitigate climate change via CO_2_ reutilisation. In this context, strategies such as CO_2_ methanation and reverse water-gas shift (RGWS) has been targeted as a suitable approach to reutilise the CO_2_ (Saeidi et al., 2014; Ghaib et al., 2016).

CO_2_ chemistry has become one of the most important branches of chemistry because it is a non-toxic, abundant, and renewable C_1_ resource. Particularly, the use of CO_2_ instead of harmful CO in the synthesis of crucial chemicals has recently attracted a lot of interest. The RWGS reaction is the key step for these kinds of processes, and derived from this reaction, various valuable chemicals have been produced from CO_2_ instead of CO (Daza and Kuhn, 2016).

Because of the inertness of CO_2_ and the thermodynamic limitations of the reaction, the RWGS reaction needs high temperature along with a working catalyst and the optimum reaction conditions to take place (Zhu et al., 2020).

Nowadays, many processes have been reported involved in RWGS to produce valuable compounds using CO_2_ as the C_1_ resource. For example, a two-step mechanism has been proposed for the hydrogenation of CO_2_ to chemical fuels. The first step is the reduction of CO_2_ to CO through the RWGS reaction, and the second step is the hydrogenation of CO to olefins or higher hydrocarbons through the Fischer–Tropsch synthesis (FTS). (Yang et al., 2020).

However, the operational conditions of the RWGS reaction reveal some challenges to develop this strategy. First, carbon dioxide is a highly stable molecule, and for its use as a reactant in this reaction, its high activation energy must be exceeded. The temperature of most reactions involving RWGS reaction is usually higher than 100°C owing to the inertness of CO_2_. Secondly, because of its endothermic nature, the RWGS reaction is thermodynamically favourable at high temperature. Furthermore, additional side reactions, such as CO methanation and the Sabatier reaction, would occur under similar reaction conditions, consuming significant amounts of H_2_. The design of highly efficient catalysts able to achieve low-temperature RWGS and to be highly selectivity to the desired product under mild conditions is the key to the production of important chemicals with CO_2_ through RWGS coupled with other reactions (for example, FT). (Shen et al., 2019; Yang et al., 2020) The selectivity to CO at low temperature is one of the main drawbacks of the RWGS reaction. From thermodynamics perspective, it is evident that the production of CH_4_ is highly favoured at low temperature. However, this selectivity can be altered using different strategies. For instance, selectivity of the products can be altered, 1) modifying reaction conditions (i.e., H_2_:CO_2_ ratio, catalyst amount, etc.), and 2) catalyst composition. (Smith R J et al., 2010; Frontera et al., 2017; González-Castaño et al., 2021) Metals such as Cu, Fe, Rh, Ru and Pt have been studied for the RWGS reaction showing relevant selectivity to CO (Daza and Kuhn, 2016; González-Castaño et al., 2021).

Among different metal-supported catalysts, nickel has become one of the favourite active metals to catalyse RWGS reaction due to its low cost and high catalytic activity. (Rodrigues et al., 2017; Liang et al., 2019a; Zhang et al., 2019a)Since it has been well described the effect of the support in the catalytic activity as a result of the metal-support interaction, in the specific case of Ni-based catalysts this influence has been observed in the modification of the physicochemical properties such as the dispersion of the active phase or in the reducibility of the oxide precursor. Ni has been supported in materials such as Al_2_O_3_, (Aksoylu et al., 1996; Muroyama et al., 2016), SiO_2_, (Aziz et al., 2014; Chen et al., 2017), zeolites, (Graça et al., 2014; Westermann et al., 2015), CeO_2_, (Liu et al., 2016; Varvoutis et al., 2020), and ZrO_2_ (Lu et al., 2014; Zonetti et al., 2014) observing a performance completely different in terms of conversion and products selectivity. For instance, CeO_2_ support has demonstrated to enhance the catalytic activity in Ni-CeO_2_ catalyst due to its oxygen storage capacity, which can be reversible modified depending on the experimental conditions. (Yao and Yao, 1984; Liu et al., 2012) However, even that Ni poses a relevant catalytic activity, it is well known that this transition metal presents serious drawbacks such as rapid sintering and coke formation (Charisiou et al., 2018).

In order to overcome the above-mentioned limitations and enhance activity and the CO selectivity to this low-temperatures of Ni-based catalysts for RWGS, using promoters such as noble metals, alkali, and alkaline Earth metals is a promising strategy. (Lee et al., 2021) In this sense, alkali metals are largely abundant and economically viable. Moreover, it has been demonstrated that these metals modify the physicochemical properties of Ni-based catalysts due to increase of support basicity and introducing oxygen vacancies which are essential to the initial chemisorption of CO_2_. (Liu et al., 2020) For instance, T.A. Le *et al.* reported the catalytic activity of Ni supported on SiO_2,_ and CeO_2_ doped with Na (0.1-1 wt%) observing a direct effect of the Na promoter in the catalyst and a decrease in the catalytic activity, especially in the Ni supported CeO_2_ catalyst, due to the promoter-support interaction that promotes a decrease in the catalytic active surface area. (Le et al., 2018) Furthermore, as reported elsewhere, Na addition hampers CH_4_ production while boosting the CO selectivity as a result of the lower hydrogen coverage of the active metal and the CO dissociation, which are the key steps in the CO_2_ conversion (Beierlein et al., 2019).

The effect of potassium as a promoter on the catalytic properties in Ni-based supported on alumina catalyst has been studied, showing a negative effect on the CO_2_ methanation but increasing the resistance to coking. (Tsiotsias et al., 2020) However, it has been reported the boosting effect of CO selectivity over CH_4_ in the CO_2_ methanation is mainly attributed to the weak interaction of CO and the surface catalyst. (Büchel et al., 2014) For instance, Zhang *et al.* studied the KOH doped Ni-Al_2_O_3_ catalyst in the CO_2_ methanation, which evidenced the strong interaction between the surface of the catalyst and formate intermediate; this interaction did not allow their further hydrogenation to CH_4_ and instead led to a high CO selectivity. (Zhang et al., 2019b) Moreover, different studies have suggested a direct effect between the CO_2_ conversion and the alkali promoter/active metal ratio, which makes essential the screening for the determination of the optimal promoter loading for each specific experiment configuration (i.e. catalytic system and reaction conditions) (He et al., 2014; Petala and Panagiotopoulou, 2018).

In this work, we pursue a low-temperature RWGS reaction strategy. Based on this, we aim to optimise K loading as a key parameter in a Ni/CeO_2_ catalyst and we analyse its impact on the physicochemical properties of the catalyst as well as in the catalytic activity and product selectivity. The experimental results gathered in catalytic activity tests are then validated with an Aspen-Plus process model. The overriding goal of this study is to develop advanced catalysts for a low-temperature RWGS reaction that can be integrated into complex CO_2_ utilisation schemes to foster the transition towards a low-carbon future.

## Experimental Methodology and Techniques

### Synthesis of the Catalysts

The catalysts were synthesised using a co-impregnation method based on procedures reported elsewhere. In a typical synthesis, the necessary amount of Ni(NO_3_)_2_·6H_2_O to obtain 10 wt% of Ni was mixed with the proper amount of KNO_3_ to ensure a Ni:K molar ratio of 10:0.5, 10:1 or 10:2 and dissolved in water and mixed with a commercial CeO_2_ (Rhone-Poulenc) and kept under agitation for 4 h at room temperature. Subsequently, the water was evaporated from the dissolution using dynamic vacuum in a rotary evaporator (BUCHI, Switzerland). The final material was dried a 100°C overnight prior to the calcination in air at 550°C for 4 h. For clarity, the catalyst will be label as 0, 0.5, 1 and 2K depending on the K content.

### X-Ray Diffraction (XRD) Measurements

The crystalline structure and the phase identification of the fresh, reduced and spent catalysts were elucidated using powder X-ray diffraction measurements in a diffractometer Siemens D-500 equipped with Ni-filtered Cu Kα (40 mA, 45 kV) in the 2Q range of 10° to 80° using a step time of 300 s and 0.05° as step size.

### Scanning Electron Microscopy (SEM)

The morphology and metal dispersion of the catalysts was evaluated using scanning electron microscope HITACHI S4800 equipped with cold cathode field emission gun with voltage from 0.5 to 30 kV, resolution of 1 nm at 15 kV and equipped with a Bruker-X Flash-4010 EDX detector with a resolution of 133 eV (at the MnKα line), and a detector with sample holder to work in transmission mode (STEM in-SEM).

### Nitrogen Isotherms at −196°C

The textural properties of the synthesised catalyst were evaluated using nitrogen adsorption-desorption isotherms at −196°C in a Micromeritics ASAP 2010 instrument. The specific surface area was calculated using the Brunauer-Emmett-Teller (BET) method. The average pore size distribution was obtained by the Barret-Joyner-Halenda (BJH) method and the pore volume using DR method. Prior to each experiment, the samples were outgassed at 150°C overnight under dynamic vacuum.

### Hydrogen Temperature-programmed Reduction (H_2_-TPR)

The reducibility of the catalyst was evaluated using hydrogen temperature-programmed reduction (H_2_-TPR) measurements. In a typical experiment, 50 mg of the catalyst was placed in a U-shaped reactor and exposed to a flow of 50 ml/min of mixture H_2_/Ar (5% H_2_) from room temperature to 900°C with a heating rate of 10°C/min. The H_2_ consumption was tracked using a thermal conductivity detector TCD.

### X-Ray Photoelectron Spectroscopy (XPS)

Surface chemical properties of the catalysts were evaluated by X-ray photoelectron spectroscopy (XPS) using a K-ALPHA spectrometer (Thermo Fischer Scientific) operated in constant energy mode with energy scans from 50 to 200 eV in order to measure all the whole energy band. All XPS spectra were recorded using an Al-Kα radiation (1,486.6 eV) with a twin crystal monochromator and a focused X-ray spot at 3 mA × 12 kV. Prior to the sample measurements, the samples were H_2_ reduced *ex-situ* at 750°C and conserved in octane until analysis. Before recording the spectrum, the samples were maintained in the chamber until a residual pressure of ca. 5 × 10^−7^ N/m^2^ was reached.

### Catalytic Activity

The catalytic activity of the synthesised catalysts was performed in a continuous flow fixed bed reactor system commercialised by PID. In a typical experiment, 200 mg of the catalysts were placed in the middle part of the reactor and pre-reduced (in the case of activated samples) before the experiment at 750°C for 1 h under a flow of 100 ml/min H_2_/N_2_ (50/50). After the system reached room temperature, the catalyst was exposed to 50 ml/min of gas mixture flow (50% N_2_, 40% H_2_, and 10% CO_2_). The reaction was evaluated from 200°C to 750°C with a heating rate of 10 ºC/min. All the runs were performed at atmospheric pressure and weight hourly space velocity (WHSV) was 15,000 ml/g^−1^ h^−1^ with H_2_/CO_2_ ratio of 4:1. The product outlet was analysed online using a micro-GC Varian 4,900 instrument equipped with two Porapak Q and MS-5A columns. The conversion of CO_2_ (Eq. 1) and the selectivity of CH_4_ (Eq. 2) and CO (Eq. (3)) was calculated using the following equations.

$$X_{{\mathrm{CO}}_{2}}\left(\%\right)=\frac{{\left[{\mathrm{CO}}_{2}\right]}_{\mathrm{in}}-{\left[{\mathrm{CO}}_{2}\right]}_{\mathrm{out}}}{{\left[{\mathrm{CO}}_{2}\right]}_{\mathrm{in}}}\times 100$$ (1)

$$S_{\mathrm{CH}4}\left(\%\right)=\frac{{\left[{\mathrm{CH}}_{4}\right]}_{\mathrm{out}}}{{\left[{\mathrm{CO}}_{2}\right]}_{\mathrm{in}}-{\left[{\mathrm{CO}}_{2}\right]}_{\mathrm{out}}}\times 100$$ (2)

$$S_{\mathrm{co}}\left(\%\right)=\frac{{\left[\mathrm{CO}\right]}_{\mathrm{out}}}{{\left[{\mathrm{CO}}_{2}\right]}_{\mathrm{in}}-{\left[{\mathrm{CO}}_{2}\right]}_{\mathrm{out}}}\times 100$$ (3)

where [CO_2_]_in_ is the initial concentration of CO_2_ in the inlet mixture, and [CO_2_]_out_, [CH_4_]_out_, and [CO]_out_ are the outlet concentrations of CO_2_, CH_4_, and CO, respectively.

### Thermodynamic Simulation

Aspen Plus V 8.8 package was utilised to develop a process model for seek of understanding the potential connection of our lab-scale catalytic data and simulated process for CO_2_ conversion. Thermodynamic limits of both CO_2_ methanation and RWGS reactions over a range of temperatures were examined. As comparative parameters in this validation process were also selected the conversion of CO_2_ (Eq. 1) and the selectivity of CH_4_ (Eq. 2) and CO (Eq. 3).

The process was modelled in Aspen Plus®. Chemical species involved in the model were water, methane, carbon dioxide, carbon monoxide, and hydrogen. Carbon formation or Coke formation was neglected in the modelling process as we did not observe relevant cocking on the experimental studies. The thermodynamic method used was RKSMHV2 based on the Soave-Redlich-Kwong equation. (Er-rbib and Bouallou, 2013; Adelung et al., 2021) The choice of catalyst used resulted from conclusions gathered for experimental tests. The material flows as well as pressure and temperature profile in the reactor were analogous to experimental tests. From the kinetics point of view, it was based on kinetics developed by Er-rbib (2013) and König (2014), which considered the CO_2_ methanation and RWGH reactions, respectively, using catalysts was based on nickel as this work.^39–41^The kinetic rate equations are as follow:

For Reaction 1 (Eq. 4) and Reaction 2 (Eq. (5)): (Er-rbib and Bouallou, 2013; König et al., 2015; Sun et al., 2017)

$${\text{R}}_{1}={\text{ k}}_{1}*\frac{{\text{p}}_{{\text{H}}_{2}}^{0.5}*{\text{p}}_{{\text{CO}}_{2}}^{0.5}*\left(1-\frac{{\text{p}}_{{\text{CH}}_{4}}-{\text{p}}_{{\text{H}}_{2}}^{2}}{{\text{p}}_{{\text{H}}_{2}}^{4}*{\text{p}}_{{\text{CO}}_{2}}*{\text{K}}_{\text{eq}}}\right)}{{\left(1+{\text{K}}_{\text{OH}}*\frac{{\text{p}}_{{\text{H}}_{2}\text{O}}}{{\text{p}}_{{\text{H}}_{2}}^{0.5}}+{\text{K}}_{{\text{H}}_{2}}*{\text{p}}_{{\text{H}}_{2}}^{0.5}+{\text{K}}_{\text{mix}}*{\text{P}}_{{\text{CO}}_{2}}^{0.5}\right)}^{2}}\left(\text{mol}{\left({\text{kg}}_{\text{cat}}*\text{s}\right)}^{-1}\right)$$ (4)

$${\text{R}}_{2}=\frac{{\text{k}}_{2}*{\text{K}}_{\text{c}}*{\text{P}}_{\text{CO}}^{0.5}*{\text{P}}_{{\text{H}}_{2}}^{0.5}}{{\left(1+{\text{K}}_{\text{c}}*{\text{P}}_{\text{CO}}+{\text{K}}_{\text{OH}}*{\text{P}}_{{\text{H}}_{2}\text{O}}*{\text{P}}_{{\text{H}}_{2}}^{-0.5}\right)}^{2}}\left(\text{mol }{\left({\text{kg}}_{\text{cat}}*\text{s}\right)}^{-1}\right)$$ (5)

where Rate constants K_1_ (Eq. 6) and K_2_ (Eq. 7) for the above equations were defined as function of temperature:

$${\text{k}}_{1}=6.071*10^{10}\left(\text{Pa}*\text{K}*{\text{g}}_{\text{cat}}*\text{s}\right)$$ (6)

$${\text{k}}_{2}=3.34*10^{6}*\exp\left(-\frac{74000}{\text{RT}}\right)\left(\text{Pa}*\text{K}*{\text{g}}_{\text{cat}}*\text{s}\right)$$ (7)

**FIGURE 1:**
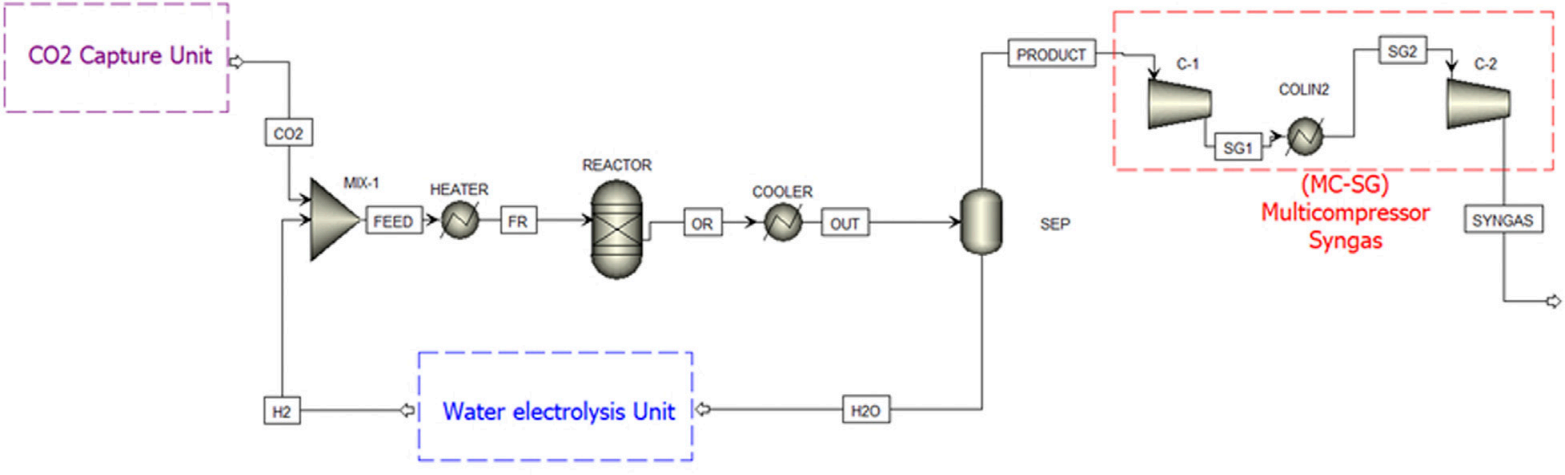
Flowsheet of the thermodynamic simulation in Aspen Plus® including compression units (MC-SG), split block (SEP) and heat exchange systems.

Figure 1 depicts the block flow diagram of the simulated process whose aim is to integrate a RWGS unit with a syngas processing reactor (i.e. Fischer-Tropsch). p = Plant size (Table 1) was simulated to reproduce laboratory scale experiments, in which two feed streams were considered. One of these was composed by CO_2_ and N_2_, which could come from a CO_2_ Captured Unit in a hypothetical real flue gas situation. The second feed stream was composed by pure H_2_, which could come from water electrolysis. In this study, the concentration, pressure and feed flows were kept constant, while the reactor operating conditions were varied from 200 to 750°C replicating the experimental study.

**TABLE 1:** Blocks description used in the thermodynamic simulation (Adelung et al., 2021).

Block ID	Aspen plus name	Description
Mix-01	Mixer	Mixes streams together
Reactor	RGibbs	Equilibrium reactor; models single phase chemical equilibrium by minimising Gibbs free energy
Heater	Heater	Heater-used to heat entrance
Cooler	Heater	Cooler-cools downs the product gas temperature to 5 C
Flash	Sep	Separator- separates water from the produced gas
Colin2	Heater	Cooler-cools down the product gas
MC-SG	Multicompressor syngas	Equipment is implemented as 2-staged compressors (C-1 and C-2) with an isentropic efficiency of 72% and 100% mechanical efficiency

Firstly, the feed streams were mixed in “*MIX-1*” before being heated to the reactor operating temperature through the block “*HEATER*”. After that, the outlet stream “*FR*” was introduced to the reactor. In this work, the Gibbs model block was used in the modelling, which assumed thermodynamic equilibrium at the respective outlet temperature and pressure of the reactor (1 bar). As shown in Figure 1, the stream “O*R*” from the reactor was cooled in block “*COOLER*” to remove most of the water because this might interfere with further processing downstream. The outlet stream “*OUT*” from “*COOLER*” was fed to the separator “SEP”, where water is separated out from the stream “*PRODUCT*”. The temperature of both blocks was set to 5°C, (Whitlow and Parrish, 2003) being the water recycled to produce hydrogen via electrolysis again.

The stream containing H_2_, N_2_, CH_4_, CO and CO_2_ were represented as “*PRODUCT*”. This stream went to the multi-stage compressor block (MC-SG), which is maintained at 230°C and comes out as the stream “*SYNGAS*”. The specification condition established was to obtain a product ready to feed a Fischer-Tropsch synthesis unit to produce liquid fuels. In this case, the model used in this study proposed exit operating conditions of 230°C and 12 bar.

## Results and Discussion

### XRD Measurements

**FIGURE 2:**
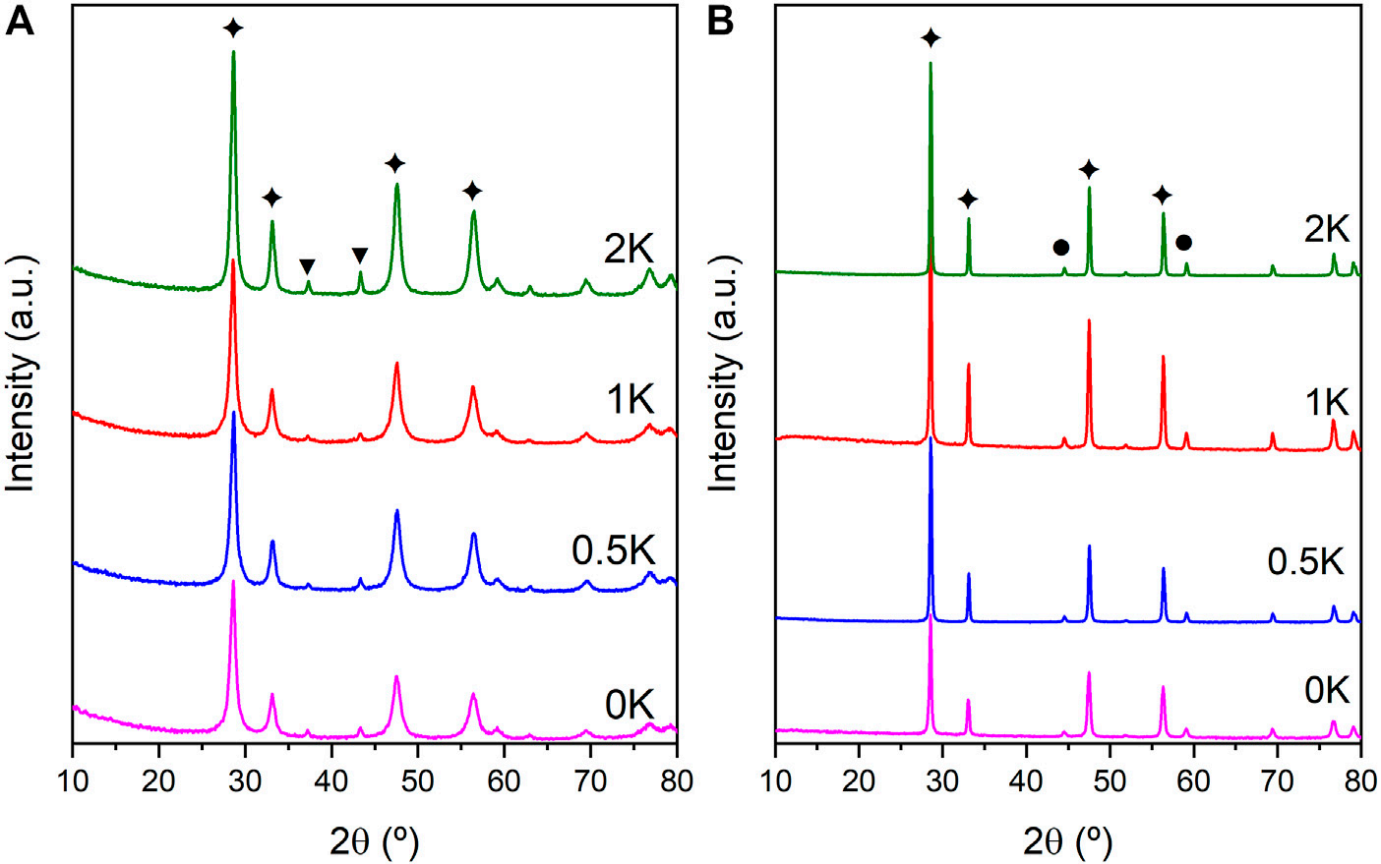
XRD patterns of the **(A)** non-reduced catalysts and **(B)** reduced catalysts. (✦ CeO_2_ JDPS 34-0,394, ● Ni JDPS 04-0,850, ▼ NiO JDPS 47-1,094).

The structural features of the synthesised catalysts were elucidated using powder XRD analysis. As it is observed in Figure 2A, the XRD pattern of the calcined Ni-based catalyst and the K-doped systems show the typical peaks attributed to crystalline CeO_2_ (JDPS 34-0,394). Furthermore, planes (111), (200) and (220) of the NiO fcc phase are observed at 37.2°, 43.3°, and 62.9°, respectively, in agreement with JCPDS no. 04-0,835. For the reduced samples, bigger particles of CeO_2_ are obtained after the reduction treatment (Figure 2B), and the typical metallic Ni reflections [(111) and (200) reflection planes) accounting for the Ni reduction during the catalyst’s activation appear in these samples at 44.5° and 51.9° (JCPD no. JDPS 04-0,850). Due to the low amount of K loaded into the catalysts and possibly to the high dispersion, any crystalline phase of this alkali metal is observed in any sample. The average crystal size of CeO_2_, NiO and Ni particles was calculated using the Scherrer equation, and the values are summarised in Table 2. It is observed an increase in the CeO_2_ crystal size of the reduced samples with the K addition, being less notorious for the 1K sample. That increase could be explained by the K incorporation to the CeO_2_ lattice. However, the ionic radius of K^+^ (0.151 nm) is much larger than that of Ce^4+^ (0.097 nm) but similar to Ce^3+^ (0.114 nm), on this basis, part of K is likely to be deposited on the surface of CeO_2_ as K_2_O and part could be expected to undergo ionic substitution in the CeO_2_ lattice (Ang et al., 2014).

**TABLE 2:** Textural properties of the catalysts.

Catalyst (K)	Crystal size (nm)				Ni amount (%)^d^	K Amount (%)^d^	S_BET_ (m^2^/g)	V_T_ (cm^3^/g)
	Fresh non-reduced		Fresh reduced					
	NiO^a^	CeO_2_ ^c^	Ni^b^	CeO_2_ ^c^				
0	17.6	16.4	18.9	27.9	10.11	—	62	0.066
0.5	24.5	14.8	24.04	36.99	9.62	0.42	24	0.049
1	17.5	18.5	28.3	34.9	8.97	0.65	42	0.051
2	30.6	14.8	24.04	36.99	9.17	1.62	26	0.054

a Calculated with NiO (200) diffraction plane.

b Calculated with Ni^0^ (111) diffraction plane.

c Calculated with CeO_2_ (111) diffraction plane.

d Obtained from EDX quantitative analysis.

Furthermore, NiO and Ni particles also suffer a size increase as an effect of the incorporation of the promoter, thus giving evidence of possible interactions of the Ni species and/or CeO_2_ with the promoter. However, in the 1K catalyst, the NiO particle is similar to the monometallic catalyst, but after the reduction process, the K-promoted catalyst suffers a slightly more considerable increase if we compare it with the monometallic counterpart. Thus, it could give clear evidence of the metal-promoter interactions, which is enhanced depending on the amount of the promoter. For instance, Borowiecki *et al.* have reported the effect of alkali metals in Ni-based catalyst describing this increment in the Ni particle size as an effect of the chemistry surface modification that enhances the particle growth. This work discusses the domination of potassium–nickel interactions for a model catalyst versus an impregnated nickel catalyst where the potassium–alumina (support) interaction dominates. (Borowiecki et al., 2014) Moreover, Chen *et al.* reported that in the pre-treatments for calcination and reduction, the addition of K species weakens the interaction between Ni^2+^ and the support enhancing the Ni particles growth. (Chen et al., 2010) Hence, it is essential to evaluate how the amount of promoter affects these interactions since it has been reported to change as a function of the promoter quantity in the system.

**FIGURE 3:**
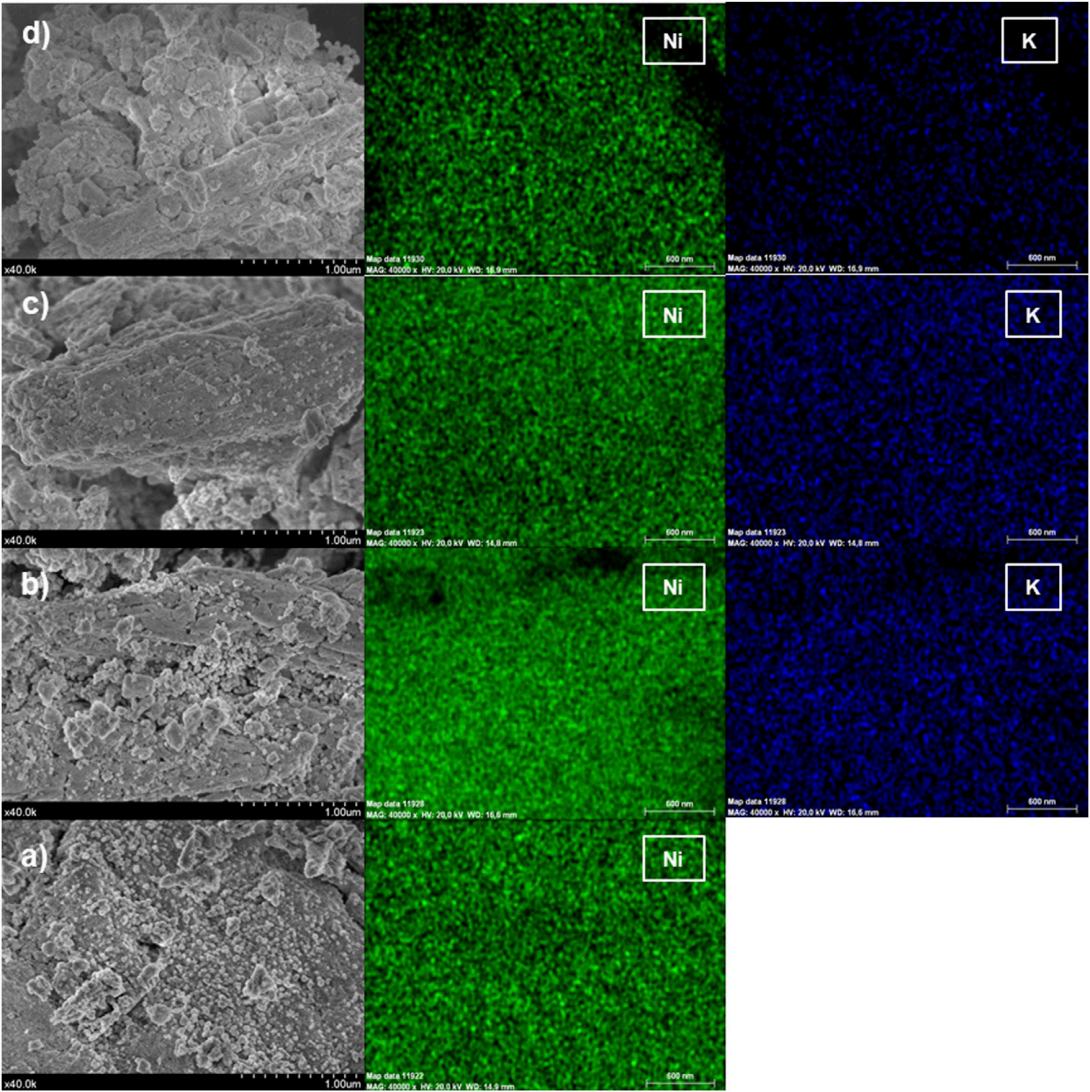
SEM images of the **(A)** 0K, **(B)** 0.5K, **(C)** 1K, and **(D)** 2K catalysts.

The morphological characteristics of the synthesised catalyst were evaluated using SEM. As observed in Figure 3, the catalysts present a heterogeneous morphology with different particles size. EDS mapping was performed in the Ni-based catalyst and its K promoted counterparts, where is observed a highly homogeneous dispersion of the Ni in the catalyst surface. Additionally, in 0.5, 1 and 2K, the K present in the surface is homogeneously dispersed. From the EDX spectra was quantified the metals on the surface (Table 2), which are in good agreement with the nominal values for Ni (10%) and K (0.37 wt%, 0.73 wt%, and 1.47 wt%), which confirm the incorporation of Ni and the promoter during the co-impregnation method.

### Nitrogen Isotherms at −196°C

**FIGURE 4:**
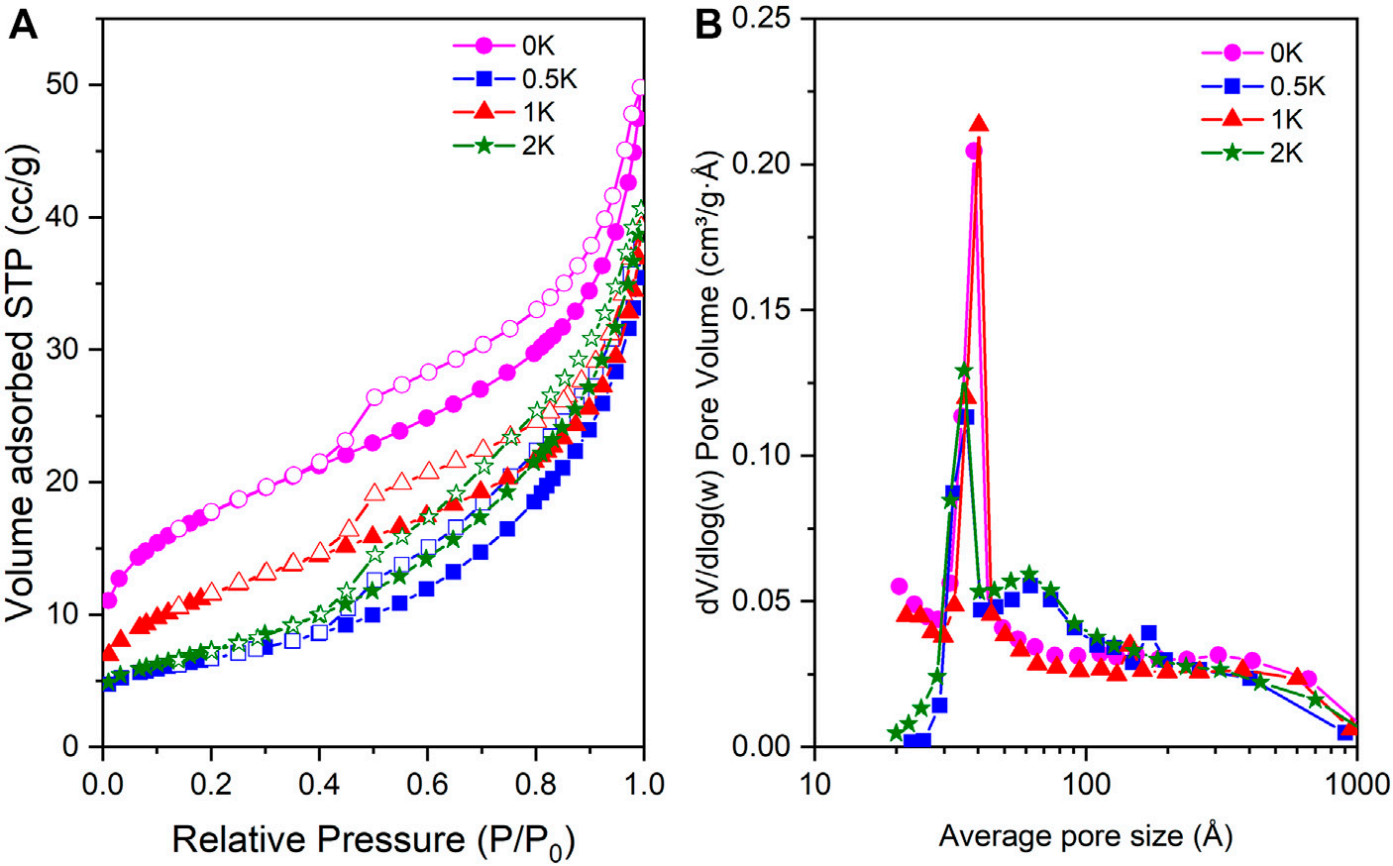
**(A)** Nitrogen adsorption-desorption isotherms at −196°C. (Filled symbols = adsorption and empty symbols = desorption) and **(B)** Pore size distribution.

The textural properties of the catalyst were evaluated using nitrogen at −196°C as a probe molecule. Figure 4A show the nitrogen adsorption-desorption isotherms of the studied catalyst. As observed, all the samples present isotherm type IV, which correspond to mesoporous materials as is confirmed in the catalysts pore size distribution (Figure 4B). Additionally, the hysteresis loop is typical of mesoporous material with H3 pore shape. (Thommes et al., 2015) As it is observed in Table 2, the surface area of the 0K catalyst is 62 m^2^/g. However, after the incorporation of the promoter, a reduction in the specific surface area is observed as a result of the increase of the particle size and to the partial blockage of the cavity after the incorporation of the K in the catalysts. Finally, the total pore volume follows a similar behaviour in the K-promoted catalysis suffering a reduction due to the incorporation of K. For instance, the decrease in the surface area of the 1K catalyst is lower, which is in good agreement with the particle size summarised in Table 2 that evidenced smaller particles size as compared with the other dopped catalysts.

### H_2_-TPR

**FIGURE 5:**
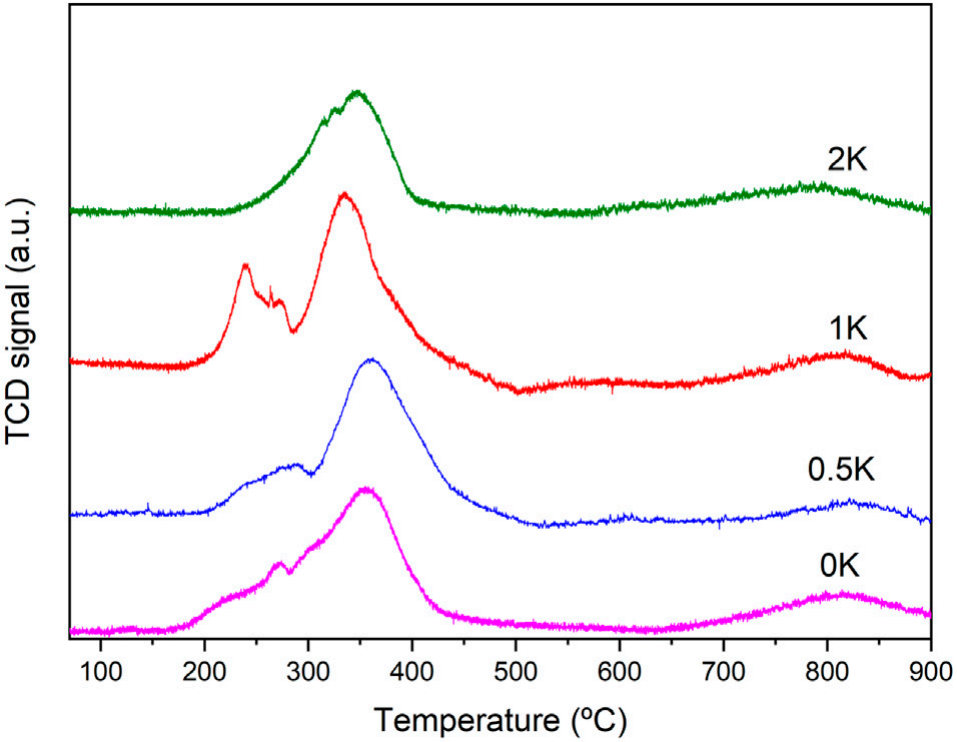
H_2_-TPR profiles of the synthesised catalysts.

The reducibility of the different species and the effect of the incorporation of K in the catalyst was evaluated using H_2_-TPR measurements. Figure 5 show the H_2_ consumption profiles of the catalyst. The high-temperature TPR reduction zone, common for all catalysts, is attributed to the reduction of bulk ceria. As observed, the 0K catalyst shows a wide region between 200 and 400°C attributed to various events, the reduction of NiO of different particle sizes and the reduction of surface ceria with different Ni-Ce interactions. However, upon promoter incorporation in the catalyst, we observed a splitting of the H_2_ consumption peaks, which is an indicator of the new interactions caused by the addition of the promoter.

The addition of the promoters can increase the reducibility due to the incorporation of alkali cations in the CeO_2_ lattice, creating oxygen vacancies and increasing the overall oxygen mobility and, by consequence, enhancing the reducibility NiO particles. (Shan et al., 2003; Ang et al., 2014) Furthermore, these reduction events can also be attributed to the interaction alkali promoters-nickel particles that facilitate the reduction of Ni due to the nature of the alkalis.

Once again, these new interactions are more notorious for the 1K sample with a higher H_2_ consumption at lower temperatures, corroborating the enhanced interaction promoter-Ni-Ce for this sample as previously discussed. Moreover, it is well known that the particle size is directly correlated with the reduction temperature. For this sample, a smaller crystallite size of NiO was obtained in the calcined catalyst, which may also contribute to the explanation in the shift to the reduction temperature of the K-promoted sample evidencing fair agreement XRD-TPR data.

### XPS

**FIGURE 6:**
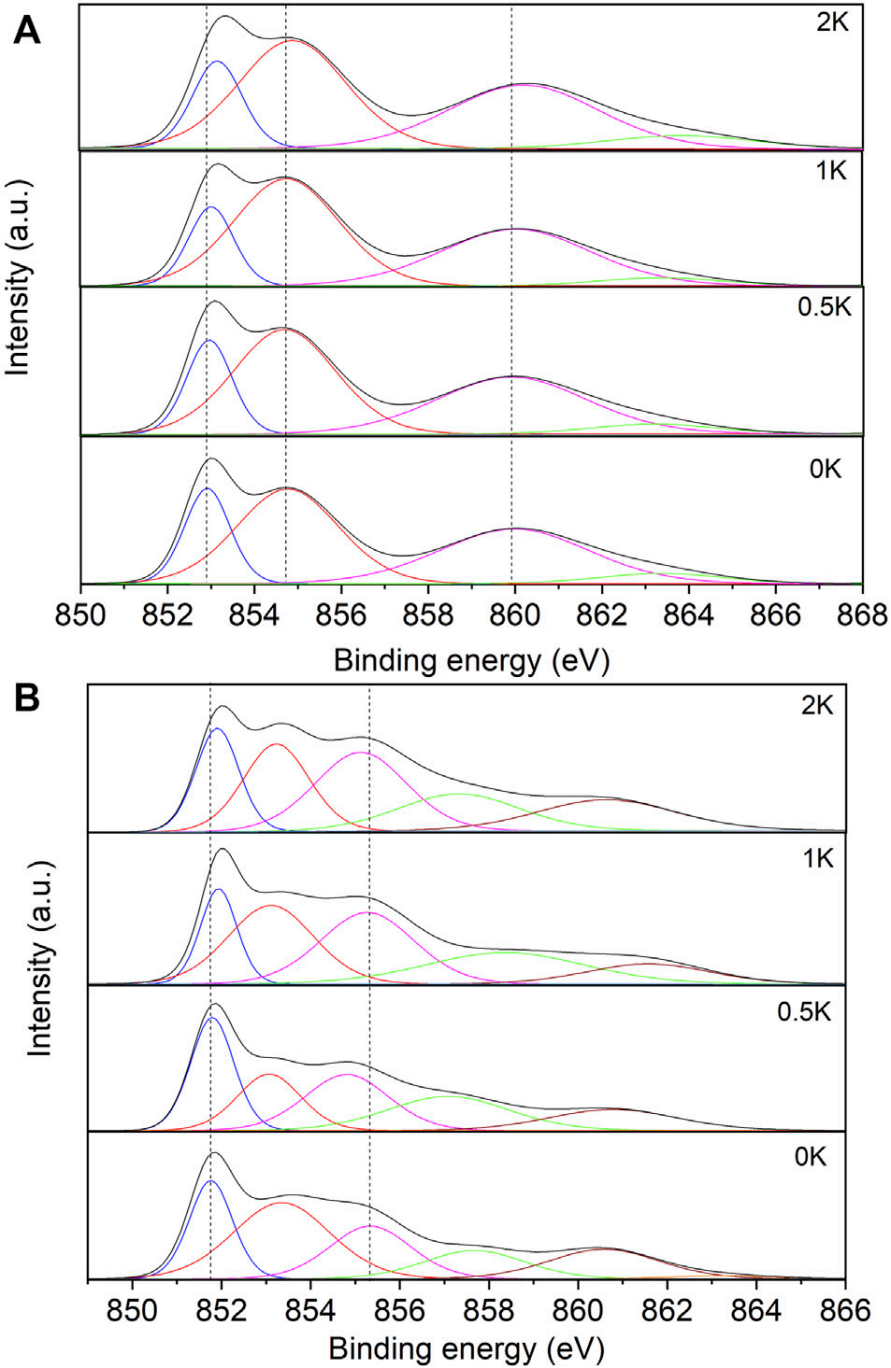
XPS spectra of the Ni 2p_3/2_ region for **(A)** the non-reduced catalysts and **(B)** reduced catalysts.

The effect of the promoter on the surface chemistry of the Ni/CeO_2_ catalyst was evaluated using XPS measurements. The Ni 2p_3/2_ XPS spectra of the non-pre-activated catalysts are shown in Figure 6A, and the binding energies of the Ni 2p_3/2_ levels for the non-reduced catalysts and Ni/support atomic ratios are summarised in Table 3. In the Ni 2p_3/2_ spectra it is observed the characteristic peaks of the Ni^2+^ under different environments close to 852.9 and 854.7 eV. However, after the incorporation of K into the catalysts, we observe a shift to higher values of B.E. due to the interactions between the catalysts and the promoter. (Pashalidis and Theocharis, 2000; Pasha et al., 2007) For instance, it has been reported the formation of mixed metal compounds K/Ni may be responsible for the change in the electronic environment change in the catalyst. (Luan et al., 2018) Finally, the increase in the Ni/Ce ratio (Table 3) of the K-promoted catalysts confirms the effect of K in the reducibility of the NiO, as it has been observed in the H_2_-TPR profiles.

**TABLE 3:** Binding energies of the Ni 2p_3/2_ levels for the non-reduced and reduced catalysts and Ni/support atomic ratios.

Catalyst (K)	Calcined	Reduced		
	Ni^2+^ (eV)	Ni^0^(eV)	Ni^2+^(eV)	Ni^0^/Ce
0	852.9–854.7	851.8	853.4–855.3	0.043
0.5	853.0–854.7	851.8	853.1–854.8	0.031
1	853.0–854.7	851.9	853.1–855.2	0.025
2	853.2–854.9	851.9	853.2–855.1	0.019

Similarly to the non-reduced catalysts, XPS measurements were performed in the H_2_ reduced catalysts. Figure 6B shows the Ni 2p_3/2_ spectra of the 0K, 0.5K. 1K and 2K catalysts where is observed the characteristic contributions of Ni^0^ at B.E. of 851.8 eV, but also the peaks attributed to the remained Ni^2+^ under different environments (853.4 and 855.3 eV) are observed. However, these peaks of Ni^2+^ corresponding to promoter samples suffer a slightly shit to lower B.E. due to the donation of a fraction of their valence electrons to the metal. (Pasha et al., 2007) Such electron donation is another evidence of a strong dopant-catalysts and metal-support interactions. The metal-support interface becomes an electronically rich site for activation of reactant molecules pushing forward the reaction.

### Catalytic Activity

**FIGURE 7:**
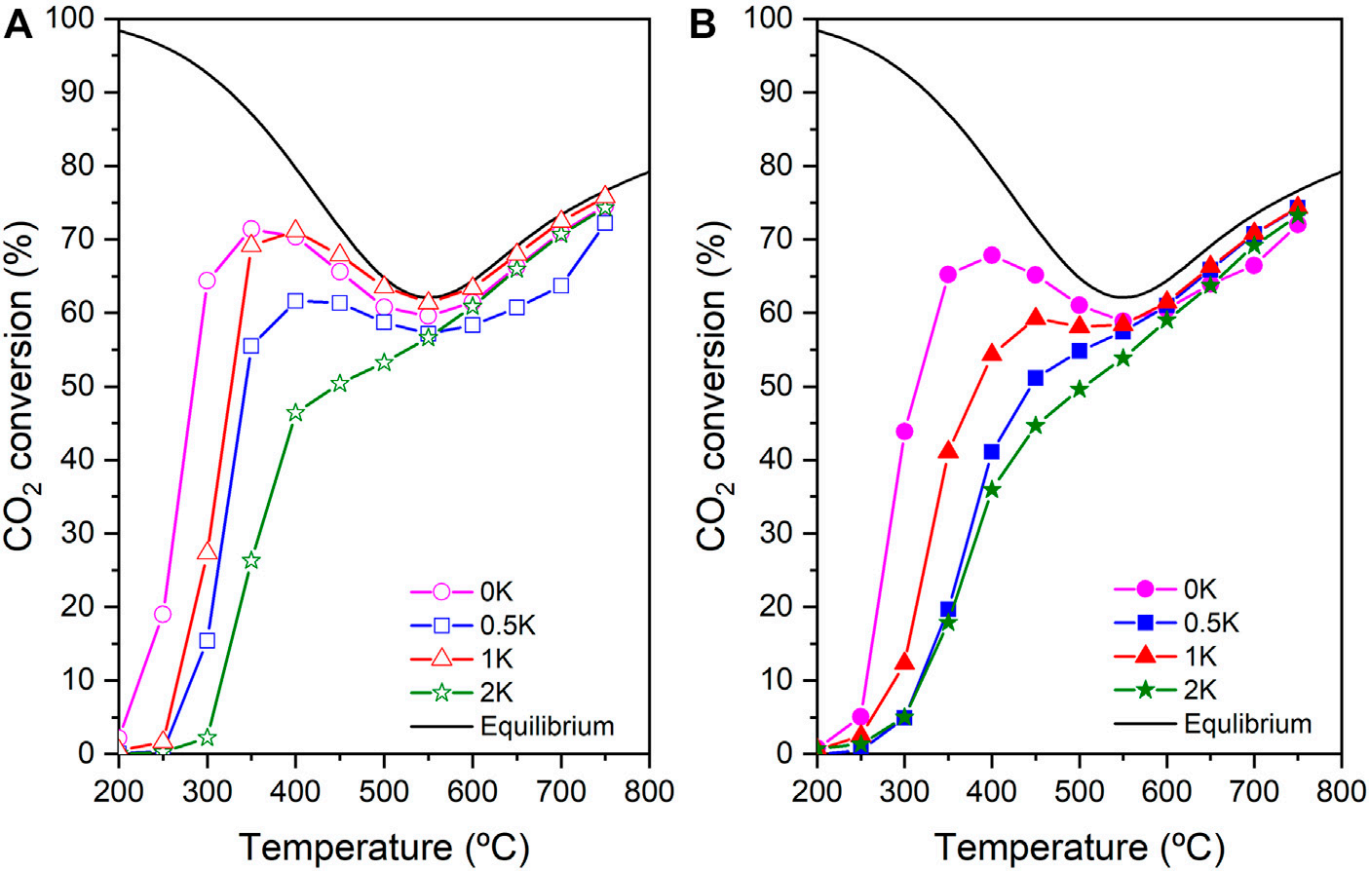
CO_2_ conversion of the **(A)** non-reduced catalysts and **(B)** reduced catalysts.

The physicochemical characterisation of the catalysts has provided evidence of relevant differences between the non-reduced catalysts and the reduced catalyst. Additionally, since H_2_ is one of the reactants in the inlet mixture, it is of high interest to evaluate the effect of the pre-activation treatment on the performance of the catalysts. Due to this, the catalytic activity of Ni/CeO_2_ and the K-promoted catalysts was evaluated under these conditions. Figures 7A,B show the CO_2_ conversion of non-reduced and reduced catalysts, respectively. In both Figures 7A,B, it is observed a negative effect on the catalytic activity as a function of the K amount in the catalyst. This decrease in the catalytic activity becomes more evident but following a similar trend for the reduced catalysts. Furthermore, C. Liang *et al.* have reported that this negative effect of alkali metal in CO_2_ conversion as a consequence of the partial coverage of Ni particles due to the promoter-Ni strong interactions. (Liang et al., 2019b) This decrease in the catalytic activity may also be attributed to the increase in the Ni particle size, as has been observed in the XRD measurements, which inherently reduces the availability of the active site. Among the K-doped samples, 1K shows the best performance than the other promoted catalysts, which confirms the optimal Ni-K interactions in this specific formulation as discussed in the characterisation section. This behaviour is also in good agreement with the H_2_-TPR profiles shown in Figure 5. Also, it must be pointed out that all catalysts tested in this study display good levels of CO_2_ conversion without pre-conditioning treatments. In other words, our catalysts are ready to work as prepared in CO_2_ conversion processes, thus saving remarkable operational costs in a potential realistic application.

**FIGURE 8:**
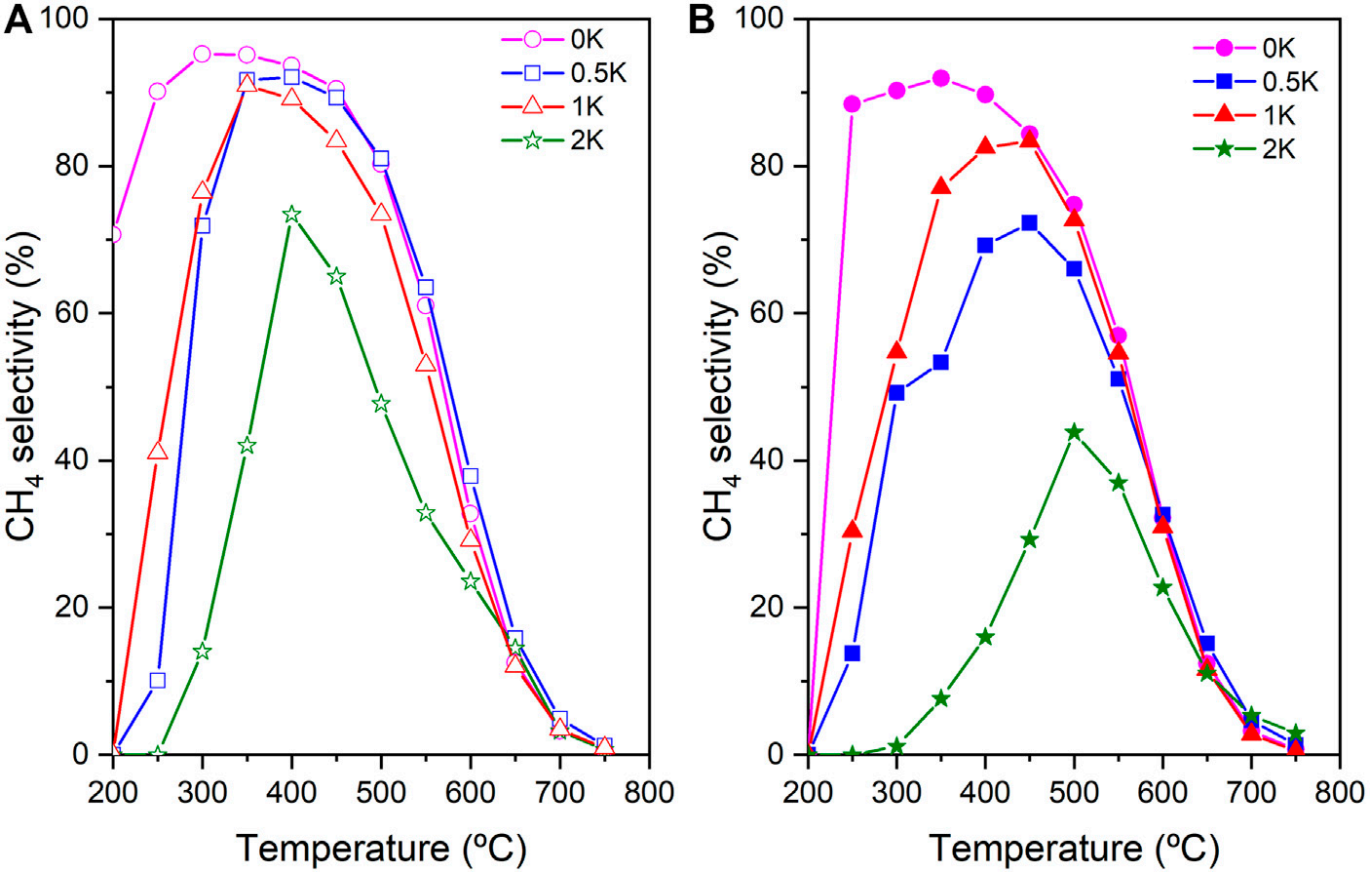
CH_4_ selectivity of **(A)** non-reduced catalysts and **(B)** reduced catalysts.

Figures 8A,B show the CH_4_ selectivity of the non-reduced and reduced catalysts, respectively. If we compare the CH_4_ selectivity of the non-promoted catalyst (0K) with the promoted counterpart (0.5, 1 and 2K), in both scenarios, it is observed suppression of the CH_4_ production as an effect of the K amount, especially in the low-temperature range which is of high interest for the low-temperature RWGS. However, this reduction becomes more evident in the pre-reduced catalysts. This reduction of the CH_4_ selectivity may be explained due to the surface chemistry modification, which promotes weak interaction CO/catalyst that restricts its further reaction to CH_4_, but also a decrease of the amount of chemisorbed hydrogen. (Beierlein et al., 2019; Vogt et al., 2019)

**FIGURE 9:**
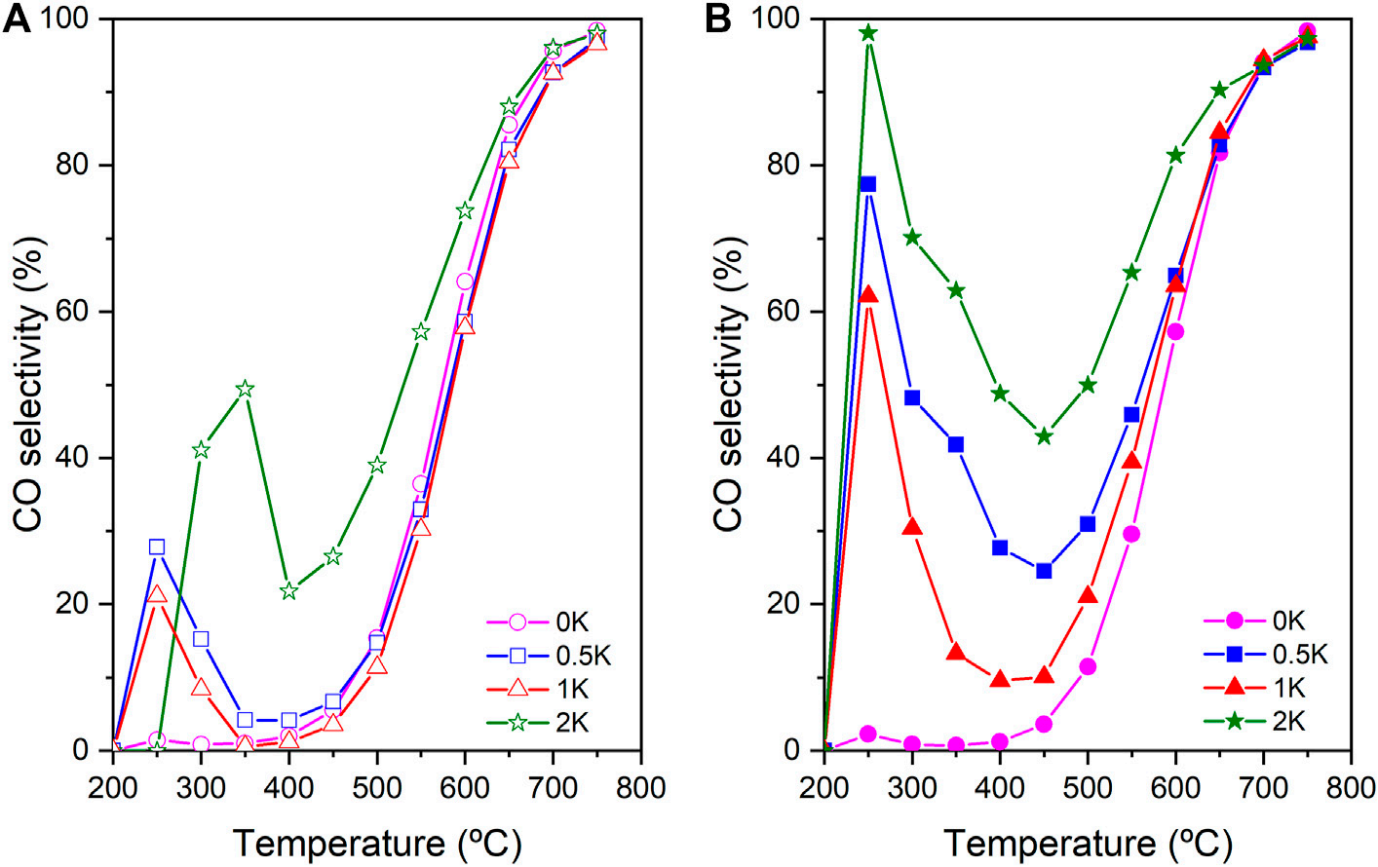
CO selectivity of **(A)** non-reduced catalysts, and **(B)** reduced catalysts.

Finally, Figure 9 shows the CO selectivity in the non-reduced (Figure 9A) and reduced (Figure 9B) catalysts where is observed a boosting effect in the production of CO as a result of the addition of the K, especially the reduced catalyst 2K that present the best CO selectivity performance at low temperature. This boosting of the CO selectivity is explained due to the modification of catalyst-CO interactions. (Ang et al., 2015; Beierlein et al., 2019) However, if we analyse the CO selectivity performance, especially in the reduced catalyst at low temperature with the CH_4_ selectivity and CO_2_ conversion, it is observed a clear enhancement of the CO at the expense of CO_2_ conversion reduction. Despite of this, a compromise must be established between CO conversion/CO_2_ conversion to potentiate the use of these novel catalysts in the RWGS reaction at low temperatures. This is achieved by elucidating the optimum amount of alkaline metal. For instance, as it has been described through this work, the 1K catalyst that has ca. 0.66 wt% of K present the best performance in terms of CO_2_ conversion compared with the 0.5 and 2K. Additionally, 1K catalyst is able to display relevant CO selectivity at low temperature.

### Thermodynamic Simulation

So far, our study demonstrated how K-doping could help us to control the CO/CH_4_ selectivity in low-temperature RWGS reactions. The question is, what would happen if we integrate these catalysts in a real chemical process for CO_2_ upgrading? To address this fundamental quest, a simplified Aspen-based process model has been developed. Figure 1 showed the block flow diagram. Our K-promoted catalysts would be implemented in the CO_2_ conversion reactor in a hypothetical application. The model allows a thorough sensitivity analysis of the operating conditions. Thermodynamic equilibrium was assumed as previously reported elsewhere. (Adelung et al., 2021) Figure 10 shows the comparison of studied parameters (conversion and selectivity) from experimental and modelling points of view in the temperature range of 200°C–750°C for K-Ni/CeO_2_ (1:10) sample. We observe a fair agreement model-experiments with very similar conversion and selectivity trends. The model predicted a slightly higher performance than the experimental data. Actually, the model marks the thermodynamic limits of the CO_2_ conversion and CO/CH_4_ selectivity. Our catalysts hit high levels of conversion very close to the maximum allowed by thermodynamics. Very importantly in the low-temperature range (i.e. 300°C) our K-doped catalysts exceeds the expectations in terms of CO selectivity, indicating the optimal role of K to suppress methanation favouring low-temperature RWGS. Additionally, the modelling-validation process was conceived to gather computerised support that allows studying the behaviour of the process evaluating other parameters such as the feed concentration or pressure. This opens a straightforward route for a forthcoming study to evaluate the process from a technical-economic perspective.

**FIGURE 10:**
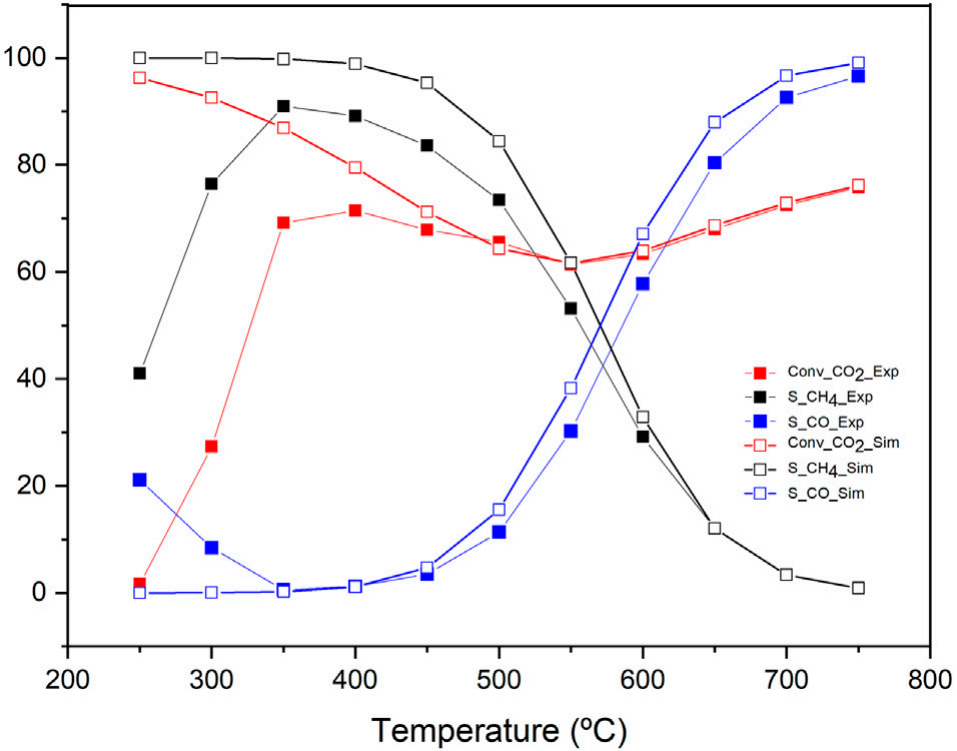
Sensitivity Analysis of the process comparing CO_2_ conversion, CH_4_ selectivity and CO selectivity from the present model and experimental results.

## Conclusion

Controlling selectivity in gas-phase CO_2_ hydrogenation reactions is a challenge in heterogeneous catalysis. This work showcases a strategy to design highly efficient low-temperature RWGS catalysts. The addition of potassium as a promoter suppresses the CO_2_-methanation in the low-temperature range at a minimal CO_2_ conversion cost. The electronic interactions K-Ni and K-Ni/CeO_2_ favour CO formation in the catalysts’ surface over methane production. Our XPS and TPR data evidence such promotional effect of K. Typically, upon increasing K loading, a greater methanation suppression is achieved. However, there is also a greater sacrifice in terms of CO_2_ conversion. Our study reveals that the 1K-doped sample presents the optimal activity selectivity trade-off.

The experimental data were contrasted with the trends obtained in a thermodynamic process modelling in an attempt to check whether our promoted catalysts would perform well in a realistic CO_2_ upgrading unit. The experimental results followed very well the modelling trends. Our catalysts reached conversion levels very close to the maximum allowed by thermodynamics, a remarkable indication of the promising behaviour of the designed materials. The combination of process simulation and lab-scale experiments provides interesting insights and open room for further techno-economic studies. Overall, this paper reveals a route to design low-temperature RWGS catalysts using non-expensive active phases evidencing the central role of catalysis and reaction engineering in the transition towards a low-carbon economy.

## Data Availability

The XRD patterns data in this study are available here: https://figshare.com/articles/dataset/XRD_patterns_Frontiers_Manuscript_785571/16847320.

## References

[B1] Adelung S. Maier S. Dietrich R.-U. (2021). Impact of the Reverse Water-Gas Shift Operating Conditions on the Power-To-Liquid Process Efficiency. Sustainable Energ. Tech. Assessments 43, 100897. 10.1016/j.seta.2020.100897

[B2] Aksoylu A. E. Akin A. N. Önsan Z. I. Trimm D. L. (1996). Structure/Activity Relationships in Coprecipitated Nickel-Alumina Catalysts Using CO2 Adsorption and Methanation. Appl. Catal. A. Gen. 145 (1–2), 185–193. 10.1016/0926-860X(96)00143-3

[B3] Ang M. L. Oemar U. Kathiraser Y. Saw E. T. Lew C. H. K. Du Y. (2015). High-temperature Water-Gas Shift Reaction over Ni/xK/CeO2 Catalysts: Suppression of Methanation via Formation of Bridging Carbonyls. J. Catal. 329, 130–143. 10.1016/j.jcat.2015.04.031

[B4] Ang M. L. Oemar U. Saw E. T. Mo L. Kathiraser Y. Chia B. H. (2014). Highly Active Ni/xNa/CeO2 Catalyst for the Water-Gas Shift Reaction: Effect of Sodium on Methane Suppression. ACS Catal. 4 (9), 3237–3248. 10.1021/cs500915p

[B5] Aziz M. A. A. Jalil A. A. Triwahyono S. Mukti R. R. Taufiq-Yap Y. H. Sazegar M. R. (2014). Highly Active Ni-Promoted Mesostructured Silica Nanoparticles for CO2 Methanation. Appl. Catal. B: Environ. 147, 359–368. 10.1016/j.apcatb.2013.09.015

[B6] Beierlein D. Häussermann D. Pfeifer M. Schwarz T. Stöwe K. Traa Y. (2019). Is the CO2 Methanation on Highly Loaded Ni-Al2O3 Catalysts Really Structure-Sensitive? Appl. Catal. B: Environ. 247, 200–219. 10.1016/j.apcatb.2018.12.064

[B7] Borowiecki T. Denis A. Rawski M. Gołębiowski A. Stołecki K. Dmytrzyk J. (2014). Studies of Potassium-Promoted Nickel Catalysts for Methane Steam Reforming: Effect of Surface Potassium Location. Appl. Surf. Sci. 300, 191–200. 10.1016/j.apsusc.2014.02.053

[B8] Büchel R. Baiker A. Pratsinis S. E. (2014). Effect of Ba and K Addition and Controlled Spatial Deposition of Rh in Rh/Al2O3 Catalysts for CO2 Hydrogenation. Appl. Catal. A: Gen. 477, 93–101. 10.1016/j.apcata.2014.03.010

[B9] Charisiou N. D. Siakavelas G. Tzounis L. Sebastian V. Monzon A. Baker M. A. (2018). An in Depth Investigation of Deactivation through Carbon Formation during the Biogas Dry Reforming Reaction for Ni Supported on Modified with CeO2 and La2O3 Zirconia Catalysts. Int. J. Hydrogen Energ. 43 (41), 18955–18976. 10.1016/j.ijhydene.2018.08.074

[B10] Chen C.-S. Budi C. S. Wu H.-C. Saikia D. Kao H.-M. (2017). Size-Tunable Ni Nanoparticles Supported on Surface-Modified, Cage-type Mesoporous Silica as Highly Active Catalysts for CO2 Hydrogenation. ACS Catal. 7 (12), 8367–8381. 10.1021/acscatal.7b02310

[B11] Chen C. S. Lin J. H. You J. H. Yang K. H. (2010). Effects of Potassium on Ni−K/Al2O3 Catalysts in the Synthesis of Carbon Nanofibers by Catalytic Hydrogenation of CO2. J. Phys. Chem. A. 114 (11), 3773–3781. 10.1021/jp904434e 19655780

[B12] Daza Y. A. Kuhn J. N. (2016). CO2conversion by Reverse Water Gas Shift Catalysis: Comparison of Catalysts, Mechanisms and Their Consequences for CO2conversion to Liquid Fuels. RSC Adv. 6 (55), 49675–49691. 10.1039/C6RA05414E

[B13] Er-rbib H. Bouallou C. (2013). Modelling and Simulation of Methanation Catalytic Reactor for Renewable Electricity Storage. Chem. Eng. Trans. 35, 541–546. 10.3303/CET1335090

[B14] Frontera P. Macario A. Ferraro M. Antonucci P. (2017). Supported Catalysts for CO2 Methanation: A Review. Catalysts 7 (12), 59. 10.3390/catal7020059

[B15] Ghaib K. Nitz K. Ben-Fares F.-Z. (2016). Chemical Methanation of CO2: A Review. ChemBioEng Rev. 3 (6), 266–275. 10.1002/cben.201600022

[B16] González-Castaño M. Dorneanu B. Arellano-García H. (2021). The Reverse Water Gas Shift Reaction: A Process Systems Engineering Perspective. React. Chem. Eng. 6 (6), 954–976. 10.1039/D0RE00478B

[B17] Graça I. González L. V. Bacariza M. C. Fernandes A. Henriques C. Lopes J. M. (2014). CO2 Hydrogenation into CH4 on NiHNaUSY Zeolites. Appl. Catal. B: Environ. 147, 101–110. 10.1016/j.apcatb.2013.08.010

[B18] He L. Lin Q. Liu Y. Huang Y. (2014). Unique Catalysis of Ni-Al Hydrotalcite Derived Catalyst in CO2 Methanation: Cooperative Effect between Ni Nanoparticles and a Basic Support. J. Energ. Chem. 23 (5), 587–592. 10.1016/S2095-4956(14)60144-3

[B19] Jetten J. Fielding K. S. Crimston C. R. Mols F. Haslam S. A. (2021). Responding to Climate Change Disaster. Eur. Psychol. 26 (3), 161–171. 10.1027/1016-9040/a000432

[B20] König D. H. Baucks N. Dietrich R.-U. Wörner A. (2015). Simulation and Evaluation of a Process Concept for the Generation of Synthetic Fuel from CO2 and H2. Energy 91, 833–841. 10.1016/j.energy.2015.08.099

[B21] Le T. A. Kim T. W. Lee S. H. Park E. D. (2018). Effects of Na Content in Na/Ni/SiO 2 and Na/Ni/CeO 2 Catalysts for CO and CO 2 Methanation. Catal. Today 303, 159–167. 10.1016/j.cattod.2017.09.031

[B22] Lee W. J. Li C. Prajitno H. Yoo J. Patel J. Yang Y. (2021). Recent Trend in Thermal Catalytic Low Temperature CO2 Methanation: A Critical Review. Catal. Today 368 (2019), 2–19. 10.1016/j.cattod.2020.02.017

[B23] Letcher T. M. (2021). “Global Warming-A Complex Situation,” in Climate Change (Amsterdam: Elsevier), 3–17. 10.1016/B978-0-12-821575-3.00001-3

[B24] Liang C. Hu X. Wei T. Jia P. Zhang Z. Dong D. (2019). Methanation of CO2 over Ni/Al2O3 Modified with Alkaline Earth Metals: Impacts of Oxygen Vacancies on Catalytic Activity. Int. J. Hydrogen Energ. 44 (16), 8197–8213. 10.1016/j.ijhydene.2019.02.014

[B25] Liang C. Ye Z. Dong D. Zhang S. Liu Q. Chen G. (2019). Methanation of CO2: Impacts of Modifying Nickel Catalysts with Variable-Valence Additives on Reaction Mechanism. Fuel 254, 115654. 10.1016/j.fuel.2019.115654

[B26] Liu H. Zou X. Wang X. Lu X. Ding W. (2012). Effect of CeO2 Addition on Ni/Al2O3 Catalysts for Methanation of Carbon Dioxide with Hydrogen. J. Nat. Gas Chem. 21 (6), 703–707. 10.1016/S1003-9953(11)60422-2

[B27] Liu K. Xu X. Xu J. Fang X. Liu L. Wang X. (2020). The Distributions of Alkaline Earth Metal Oxides and Their Promotional Effects on Ni/CeO2 for CO2 Methanation. J. CO2 Utilization 38, 113–124. 10.1016/j.jcou.2020.01.016

[B28] Liu Y. Li Z. Xu H. Han Y. (2016). Reverse Water-Gas Shift Reaction over Ceria Nanocube Synthesized by Hydrothermal Method. Catal. Commun. 76, 1–6. 10.1016/j.catcom.2015.12.011

[B29] Lu H. Yang X. Gao G. Wang K. Shi Q. Wang J. (2014). Mesoporous Zirconia-Modified Clays Supported Nickel Catalysts for CO and CO 2 Methanation. Int. J. Hydrogen Energ. 39 (33), 18894–18907. 10.1016/j.ijhydene.2014.09.076

[B30] Luan X. Yong J. Dai X. Zhang X. Qiao H. Yang Y. (2018). Tungsten-Doped Molybdenum Sulfide with Dominant Double-Layer Structure on Mixed MgAl Oxide for Higher Alcohol Synthesis in CO Hydrogenation. Ind. Eng. Chem. Res. 57 (31), 10170–10179. 10.1021/acs.iecr.8b01378

[B31] Muroyama H. Tsuda Y. Asakoshi T. Masitah H. Okanishi T. Matsui T. (2016). Carbon Dioxide Methanation over Ni Catalysts Supported on Various Metal Oxides. J. Catal. 343, 178–184. 10.1016/j.jcat.2016.07.018

[B32] National Research Council (Us) (2009). Committee on Achieving Sustainable Global Capacity for Surveillance and Response to Emerging Diseases of Zoonotic OriginNational Research Council (US) Committee on Achieving Sustainable Global Capacity for Surveillance and R. *Sustaining Global Surveillance and Response to Emerging Zoonotic Diseases* . Washington, D.C., D.C.: National Academies Press. 10.17226/12625 25032336

[B33] Pasha N. Lingaiah N. Siva Sankar Reddy P. Sai Prasad P. S. (2007). An Investigation into the Effect of Cs Promotion on the Catalytic Activity of NiO in the Direct Decomposition of N2O. Catal. Lett. 118 (1–2), 64–68. 10.1007/s10562-007-9146-1

[B34] Pashalidis I. Theocharis C. R. (2000). “Investigations on the Surface Properties of Pure and Alkali or Alkaline Earth Metal Doped Ceria,” in Studies in Surface Science and Catalysis (Amsterdam: Elsevier), 128, 643–652. 10.1016/S0167-2991(00)80070-1

[B35] Petala A. Panagiotopoulou P. (2018). Methanation of CO2 over Alkali-Promoted Ru/TiO2 Catalysts: I. Effect of Alkali Additives on Catalytic Activity and Selectivity. Appl. Catal. B: Environ. 224, 919–927. 10.1016/j.apcatb.2017.11.048

[B36] Rodrigues M. T. Zonetti P. C. Alves O. C. Sousa-Aguiar E. F. Borges L. E. P. Appel L. G. (2017). RWGS Reaction Employing Ni/Mg(Al,Ni)O − the Role of the O Vacancies. Appl. Catal. A: Gen. 543, 98–103. 10.1016/j.apcata.2017.06.026

[B37] Saeidi S. Amin N. A. S. Rahimpour M. R. (2014). Hydrogenation of CO2 to Value-Added Products-A Review and Potential Future Developments. J. CO2 Utilization 5, 66–81. 10.1016/j.jcou.2013.12.005

[B38] Shan W. Luo M. Ying P. Shen W. Li C. (2003). Reduction Property and Catalytic Activity of Ce1−XNiXO2 Mixed Oxide Catalysts for CH4 Oxidation. Appl. Catal. A: Gen. 246 (1), 1–9. 10.1016/S0926-860X(02)00659-2

[B39] Shen X. Meng Q. Dong M. Xiang J. Li S. Liu H. (2019). Low‐Temperature Reverse Water-Gas Shift Process and Transformation of Renewable Carbon Resources to Value‐Added Chemicals. ChemSusChem. 12 (23), 5149–5156. 10.1002/cssc.201902404 31605451

[B40] Smith R. J. Loganathan M. Shantha M. S. (2010). A Review of the Water Gas Shift Reaction Kinetics. Int. J. Chem. React. Eng. 8 (1), 1–49. 10.2202/1542-6580.2238

[B41] Sun L. Luo K. Fan J. (2017). Numerical Simulation of CO Methanation for the Production of Synthetic Natural Gas in a Fluidized Bed Reactor. Energy Fuels 31 (9), 10267–10273. 10.1021/acs.energyfuels.7b01781

[B42] Thommes M. Kaneko K. Neimark A. V. Olivier J. P. Rodriguez-Reinoso F. Rouquerol J. (2015). Physisorption of Gases, with Special Reference to the Evaluation of Surface Area and Pore Size Distribution (IUPAC Technical Report). Pure Appl. Chem. 87 (9–10), 1051–1069. 10.1515/pac-2014-1117

[B43] Tsiotsias A. I. Charisiou N. D. Yentekakis I. V. Goula M. A. (2020). The Role of Alkali and Alkaline Earth Metals in the CO2 Methanation Reaction and the Combined Capture and Methanation of CO2. Catalysts 10 (7), 812. 10.3390/catal10070812

[B44] Varvoutis G. Lykaki M. Stefa S. Papista E. Carabineiro S. A. C. Marnellos G. E. (2020). Remarkable Efficiency of Ni Supported on Hydrothermally Synthesized CeO2 Nanorods for Low-Temperature CO2 Hydrogenation to Methane. Catal. Commun. 142, 106036. 10.1016/j.catcom.2020.106036

[B45] Vogt C. Monai M. Kramer G. J. Weckhuysen B. M. (2019). The Renaissance of the Sabatier Reaction and its Applications on Earth and in Space. Nat. Catal. 2 (3), 188–197. 10.1038/s41929-019-0244-4

[B46] Westermann A. Azambre B. Bacariza M. C. Graça I. Ribeiro M. F. Lopes J. M. (2015). Insight into CO2 Methanation Mechanism over NiUSY Zeolites: An Operando IR Study. Appl. Catal. B: Environ. 174-175, 120–125. 10.1016/j.apcatb.2015.02.026

[B47] Whitlow J. E. Parrish C. F. (2003). Operation, Modeling and Analysis of the Reverse Water Gas Shift Process. AIP Conf. Proc. 1116 (2003), 1116–1123. 10.1063/1.1541409

[B48] Yang L. Pastor-Pérez L. Villora-Pico J. J. Gu S. Sepúlveda-Escribano A. Reina T. R. (2020). CO2 Valorisation via Reverse Water-Gas Shift Reaction Using Promoted Fe/CeO2-Al2O3 Catalysts: Showcasing the Potential of Advanced Catalysts to Explore New Processes Design. Appl. Catal. A: Gen. 593, 117442. 10.1016/j.apcata.2020.117442

[B49] Yao H. Yao Y. F. Y. (1984). Ceria in Automotive Exhaust Catalysts I. Oxygen Storage. J. Catal. 86 (2), 254–265. 10.1016/0021-9517(84)90371-3

[B50] Zhang Z. Hu X. Wang Y. Hu S. Xiang J. Li C. (2019). Regulation the Reaction Intermediates in Methanation Reactions via Modification of Nickel Catalysts with Strong Base. Fuel 237, 566–579. 10.1016/j.fuel.2018.10.052

[B51] Zhang Z. Tian Y. Zhang L. Hu S. Xiang J. Wang Y. (2019). Impacts of Nickel Loading on Properties, Catalytic Behaviors of Ni/γ-Al2O3 Catalysts and the Reaction Intermediates Formed in Methanation of CO2. Int. J. Hydrogen Energ. 44 (18), 9291–9306. 10.1016/j.ijhydene.2019.02.129

[B52] Zhu M. Ge Q. Zhu X. (2020). Catalytic Reduction of CO2 to CO via Reverse Water Gas Shift Reaction: Recent Advances in the Design of Active and Selective Supported Metal Catalysts. Trans. Tianjin Univ. 26 (3), 172–187. 10.1007/s12209-020-00246-8

[B53] Zonetti P. C. Letichevsky S. Gaspar A. B. Sousa-Aguiar E. F. Appel L. G. (2014). The NixCe0.75Zr0.25−xO2 Solid Solution and the RWGS. Appl. Catal. A: Gen. 475, 48–54. 10.1016/j.apcata.2014.01.004

